# Potential and Risks Behind the National Transformation Program in Saudi Arabia

**DOI:** 10.7759/cureus.65047

**Published:** 2024-07-21

**Authors:** Khalid Alkhurayji, Hazzam A Alzahrani, Amal s Alotaibi, Abdulaziz G Alharbi, Abdullah A Zandan, Hussein Alsheikhi

**Affiliations:** 1 Public Health, Health Information Management and Technology Department, Imam Abdulrahman Bin Faisal University, Riyadh, SAU; 2 Emergency Department, Prince Sultan Military Medical City, Riyadh, SAU; 3 Dental Center, Prince Sultan Military Medical City, Riyadh, SAU; 4 Dental Department, King Abdulaziz Naval Base Hospital, Jubail, SAU

**Keywords:** workforce, care delivery, sustainability, healthcare, reform

## Abstract

Saudi Arabia guarantees citizens the right to receive medical care and treatment during emergencies or sickness and aging. However, with the consistent increase in expenditure and inability to provide access, the transformation was an unavoidable action. Therefore, this paper aims to address the potential and risks behind the National Transformation Program (NTP) in Saudi Arabia through the lens of the Value Transformation Framework. Multiple research databases (PubMed, Web of Science, UpToDate, Google Scholar, and Summon) were searched between 2016 and 2024. Relevant articles were selected by scanning the title and abstract, yielding 34 references after the screening, exclusion, and inclusion criteria were met. Citation software was used to identify additional sources as analysis proceeded, in accordance with the hermeneutic approach in mapping and classification. The most cited concerns were the sustainability and workforce of the healthcare system. In terms of care delivery, the literature was extensive. In contrast, insufficient studies have been conducted on infrastructure and people. Furthermore, limited information is available on how to assess the transformation, which remains an unaddressed research question. NTP could meet several hurdles. However, through the measurement, assessment phases, and development tracking, success could be achieved.

## Introduction and background

The Kingdom of Saudi Arabia Constitution Law guarantees citizens the right to have medical care and treatment during emergencies or sicknesses [1]. However, for the last two decades, the expenditure per capita has risen tragically to reach a cost for each citizen from 370.42 dollars in 2000 to 1291.13 dollars in 2019 [2]. Nonetheless, the population of Saudi Arabia massively increased from 22,678,262 in 2004 to 32,175,224 in 2022 [3]. As a result, the burden on the healthcare system to address the population's needs against the increase in the incidence of mortality and morbidity rate and chronic diseases spread, in addition to the increase in life expectancy, makes the healthcare system struggle to reach the desired goals [4-6].

Delving deeper into the healthcare system, the government of Saudi Arabia previously assigned the Ministry of Health to provide free and accessible healthcare for all citizens. The Ministry of Health was responsible for managing, designing, and planning the healthcare system [4,7]. Historically, the Ministry of Health focused on the provision of medical care in the aspects of curative treatment rather than prevention [8]. However, with the consistent increase in expenditure, inability to provide access, and increased demand for healthcare, the transformation was an unavoidable action [9,10].

The National Transformation Program (NTP) is a key component of Saudi Arabia's Vision 2030, an ambitious initiative aimed at diversifying the economy and improving various sectors, including health care, to reduce the kingdom's dependence on oil revenues. Presently, the NTP was launched among 24 government bodies in 2016 with a major reform in the healthcare system. Consequently, the healthcare system reform was initiated in 2021 with the elements of value-based healthcare [11].

The Saudi healthcare system is currently challenged by a rising prevalence of chronic diseases, an aging population, and increasing mortality and morbidity rates. These factors, combined with rapid population growth, have significantly increased the demand for healthcare services and strained the existing infrastructure. The Value Transformation Framework is a method used to evaluate the effectiveness of healthcare reforms by focusing on dimensions such as care delivery, infrastructure, and workforce. It helps identify potential improvements and risks associated with large-scale transformation programs. Understanding the potential benefits and risks of the NTP is crucial for ensuring the success of the healthcare reforms. By examining these aspects, this study aims to provide insights that can guide policymakers and stakeholders in making informed decisions to achieve the goals of Vision 2030.

## Review

Methodology

**Figure 1:**
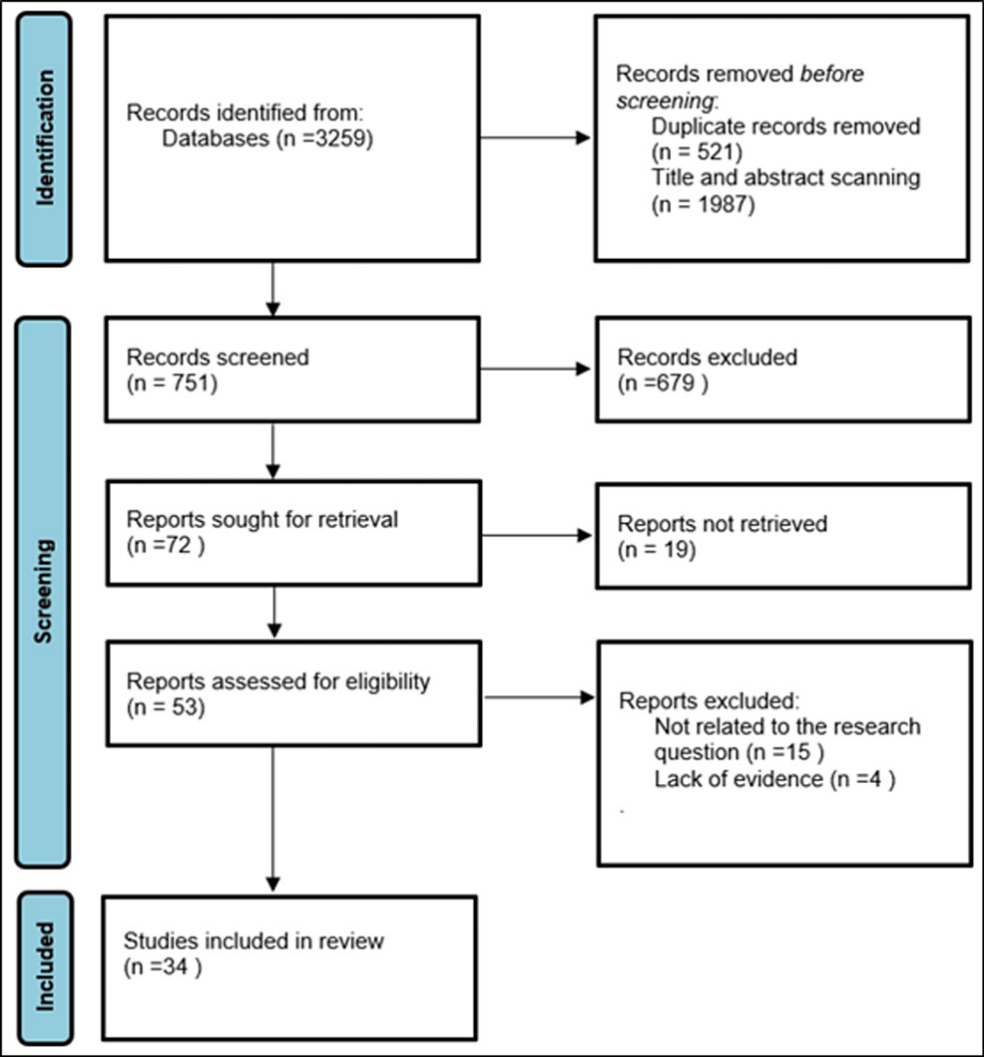
Preferred Reporting Items for Systematic Reviews and Meta-Analyses (PRISMA) flowchart outlining the study selection process.

In this review, the researchers aim to address the literature evidence using the Value Transformation Framework [12]. Moreover, Figure 1 illustrates the search strategy, which was carried out using the Preferred Reporting Items for Systematic Reviews and Meta-Analyses (PRISMA) criteria to ensure the present review's accuracy and transparency. The literature search was conducted across multiple databases, including PubMed, Web of Science, UpToDate, Google Scholar, and Summon. The search covered publications from January 2016 to June 2024. The search terms used included a combination of keywords and Boolean operators: ("risk*" OR "hazard" OR “danger*” OR “threat” OR “peril” OR “menace” OR “pitfall” OR “opportunity” OR “possib*” OR “potentials” OR “capabl*”) AND ("transform*" OR "reform*" OR "conversion" OR “transition*”) AND (“Saudi* OR “Saudi Arabia*”) [13]. A hermeneutic approach has been undertaken to answer the research question [14].

The PICO (population, intervention, comparison, and outcomes) tool [15] was used to guide and structure the research question: What are the potential and risks behind NTP in Saudi Arabia?

The hermeneutic approach is suitable for questions requiring clarification and insight through the literature on transdisciplinary synthesis [16]. Relevant articles were selected by scanning the title and abstract. Citation software was used to identify additional sources as the analysis proceeded.

Studies were included if they were published between 2016 and 2024, were in English, and focused on the healthcare transformation in Saudi Arabia Studies. Studies were excluded if they did not directly relate to the healthcare transformation, were not peer-reviewed articles, and studies not available in full text.

Studies were first screened by title and abstract. Full texts of potentially relevant studies were then reviewed to determine final inclusion based on the criteria mentioned above.

Data were extracted using a standardized data sheet. Information collected included authors, publication year, study design, key findings on potential and risks, dimensions of the Value Transformation Framework (care delivery, infrastructure, and people), and risk of bias assessment.

The risk of bias in the included studies was assessed using the Joanna Briggs Institute (JBI) critical appraisal tools (Appendix) [17]. Each study was evaluated for methodological quality based on criteria such as sample size, study design, and potential biases in data collection and analysis.

Data were synthesized using a narrative approach, structured by the dimensions of the Value Transformation Framework. Key themes and findings were summarized for each dimension (care delivery, infrastructure, and people). Tables were used to present the main results, and findings were discussed in the context of the existing literature.

Results

A PRISMA flowchart (Figure 1) was used to outline the study selection process. Initially, 3259 records were identified through database searches. After removing duplicates and screening titles and abstracts, 679 records were excluded. Full-text reviews of the remaining studies resulted in 34 studies meeting the inclusion criteria.

Tables 1, 2 demonstrate the study's findings and indicate that NTP's potential exceeds its risk. Twelve studies were given in the infrastructure dimension and 15 in the care delivery dimension of the Value Transformation Framework. However, the people dimension revealed that there were just seven studies. In terms of risk of bias assessment, the studies included in this review provided a high-quality assessment score greater than 90%, except for certain studies that provided inadequate information.

**Table 1:** Potential of the National Transformation Program within the dimensions. RoB: risk of bias assessment.

Reference	Year	Potential	Dimension	RoB
[18]	2016	Establishment of the expert committee.	Care delivery	10 (11)
[19]	2016	Collaborative effort and transformational changes are needed to drive the nursing profession toward the best outcomes.	People	10 (11)
[20]	2018	No specific framework to address management issues concerning readiness for change and adaptation.	Infrastructure	10 (11)
[21]	2018	Saudi nationals constitute less than 20% of the pharmacists employed in the Kingdom.	People	8 (8)
[22]	2018	Clinical departments utilizing e-health at an optimum level.	Care delivery	8 (8)
[23]	2019	The success of the new healthcare model in Saudi Arabia is only possible when considering the assessment of factors influencing the national prevalence of health risk factors and early detection of chronic diseases.	Infrastructure	9 (11)
[24]	2019	Reduced oil revenues, high population growth, emerging lifestyle diseases, and demands for better quality of care.	Infrastructure	10 (11)
[25]	2019	Budget allocation and investment in health system building, improving the other contributing sectors like water, sanitation, hygiene, and nutrition.	Care delivery	10 (11)
[26]	2020	Providing formal training on health policy during physicians' medical education.	People	8 (8)
[27]	2020	Experience with the pilot database could be extended to other institutions to create a national dataset that could be used to generate real-world evidence.	People	8 (8)
[28]	2020	Improvement in physician-patient communication.	Care delivery	7 (8)
[29]	2021	The reform has contributed to an increase in primary healthcare visits, patient satisfaction, enhanced coverage of rural communities, and contributed to increasing the screening rate for prevalent chronic diseases.	Care delivery	10 (11)
[30]	2021	Achievement of making health care accessible across the region with time-bound targets.	Care delivery	10 (11)
[31]	2021	Supply- and demand-side policy interventions increase productivity among Saudi health workers.	People	8 (8)
[32]	2021	A healthcare system for a transitional economy focusing on individual and social well-being during an unexpected crisis such as the COVID-19 pandemic.	Care delivery	6 (8)
[33]	2022	Training and monitoring enhance clinical empathy.	Care delivery	7 (8)
[34]	2022	Private healthcare facilities scored higher in digital health transformation indicators.	Infrastructure	8 (8)
[35]	2022	The government has laid out a roadmap with the legislative framework.	Infrastructure	10 (11)
[36]	2022	Formation of a governing central committee, development of guidelines, adoption of a decentralized implementation system and modified budget release system, development of electronic staff bank and e-recruitment system, and the introduction of virtual healthcare under the scope of the program.	Care delivery	8 (8)
[37]	2022	Ambulatory care pharmacy services in the Kingdom of Saudi Arabia to build a more robust base structure for such services.	Care delivery	10 (11)
[38]	2022	Increasing personal health insurance coverage plays a critical role in extending access to healthcare, eliminating health inequities, enhancing population health, and reducing government spending on healthcare.	Infrastructure	8 (8)
[39]	2023	More data are being generated and included in the personal health record (PHR) to ensure an accurate and comprehensive view of the patient’s health.	Infrastructure	7 (8)
[40]	2023	Establishment of a set of health governance strategies by the governmental organizations in Saudi Arabia, which comply with the seven pillars of dental governance.	Care delivery	10 (11)
[41]	2023	Healthcare professionals must understand and value the public health model to support the planned health system reforms.	People	7 (8)
[42]	2023	Implementing the new digitization and privatization initiatives (i.e., the Wasfaty program) as a result of the transformation in the healthcare sector has led to a significant reduction in healthcare expenditures and cost savings.	Care delivery	7 (8)
[43]	2023	Develop an employee engagement and satisfaction program to track the primary healthcare (PHC) providers’ levels of satisfaction.	Care delivery	8 (8)
[44]	2023	It reviews the success in engaging with local healthcare professional communities in a standardized way and the learning from previous clusters, and elaborates on emerging implementation issues and how we may overcome them and introduce the lessons learned from this journey.	Infrastructure	10 (11)
[45]	2023	Expanding healthcare infrastructure, promoting the use of technology, improving the quality of healthcare services, and emphasizing the importance of preventive healthcare. In addition, the adoption of artificial intelligence (AI) solutions can play a crucial role in transforming the healthcare system by improving efficiency, reducing costs, and enhancing the quality of care.	Infrastructure	5 (6)
[46]	2023	There is a great need for research on the management implications of digitalization by different stakeholders. Further enhancement of digital security and the strengthening of technological information systems will contribute to the universal acceptance of digital health transformation by all involved.	Infrastructure	10 (11)
[47]	2024	Including the increased number of primary care units, community home care services, outpatient services, and consultations.	Care delivery	8 (8)

**Table 2:** Risk of the National Transformation Program within the dimensions. RoB: risk of bias assessment.

Reference	Year	Risk	Dimension	RoB
[18]	2016	Estimation of five million people visiting the Saudi Arabia Hajj in 2030.	Care delivery	10 (11)
[19]	2016	Lack of human resources in hospitals increases turnover.	People	10 (11)
[48]	2017	The healthcare system is affected by the policies and regulations.	Infrastructure	9 (10)
[20]	2018	Human issues of a healthcare organization rather than paying attention only to its economic and technical dimensions.	Infrastructure	10 (11)
[21]	2018	There is an unmet need to train Saudi citizens as pharmacists and retain them in the workforce. Addressing this issue should become an important objective in Saudi Arabia's Vision for 2030.	People	8 (8)
[22]	2018	Costs and expertise of such innovative systems in information technology apart from the lack of computer and technical expertise of the hospital staff.	Care delivery	8 (8)
[49]	2020	Amendments are required in the present strategic plan for the better management of the nursing profession.	Infrastructure	7 (7)
[29]	2021	The country still faces gaps and challenges pertaining to human resources issues, cultural and lifestyle behavior, geography, intersectoral collaboration, and primary healthcare (PHC) infrastructure.	Care delivery	10 (11)
[50]	2021	Under increased pressure to build sustainable health models. Irrespective of the abundant oil-based financial resources and a relatively small population size.	Infrastructure	7 (8)
[30]	2021	Conventional approaches to health with a focus on curative health care are not sustainable or desirable in the face of the increasing burden of chronic diseases.	Care delivery	10 (11)
[31]	2021	Severe gaps in the Saudi workforce will persist and limit progress toward health system resiliency in Saudi Arabia.	People	8 (8)
[34]	2022	Tertiary hospitals scored the lowest in digital transformation readiness.	Infrastructure	8 (8)
[35]	2022	Ongoing monitoring with adjustments as this complex and multifaceted process proceeds.	Infrastructure	10 (11)
[43]	2023	Areas of the job of PHC providers that require planned reform, such as contingent reward and communication.	Care delivery	8 (8)
[51]	2023	Despite having a high level of awareness regarding the National Transformation Program in the health sector, some healthcare workers lacked a clear understanding of the institutional transformation process and the new model of care in the health sector.	People	8 (8)
[45]	2023	The need for high-quality data and the development of regulations and guidelines.	Infrastructure	5 (6)
[47]	2024	Challenges persist in terms of geographical distribution, resource allocation, and availability of pain medications, particularly opioids.	Care delivery	8 (8)

Care Delivery

The kingdom still faces several issues that must be addressed in terms of human resources, lifestyle behavior, and geographically diffuse systems [52]. Nonetheless, Aleeban and Mackey [18] assert that transformation success is vital, especially with the increased demand for Hajj. Hence, a population of five million Muslims could visit Saudi Arabia by 2030. Fortunately, healthcare governance, which includes the formation of expert committees and the monitoring of predicted models, can advance NTP succession [53].

To illustrate NTP succession, Al Khashan et al. [29] conducted a study to assess the impact of reforms in 2019. Surprisingly, the results revealed that reform contributed to a 37.5% increase in primary healthcare visits and a satisfaction rate of 4.7%. However, according to Abdulkader et al.'s [33] point of view, empathy was reported to be an important element in the improvement of care delivery and satisfaction of patients.

According to a growing body of evidence, the most common concerns for improvement reported by secondary and primary healthcare institutions, pharmacies, and dental care were costs and innovative information management systems [37,40,54,55]. To illustrate the case, Alshammary et al. [47] and Almajed et al. [56] claimed that to reform and achieve the desired outcomes, healthcare services must prioritize access and consultation, as increased demand for healthcare is unavoidable. However, Salam et al. [25] and Alkhamis et al. [30] assert that reforms will need to involve stakeholders, administration, and policymakers to improve sustainability.

To illustrate the healthcare system's sustainability, Salam et al. [32] and Alshammari et al. [42] found that digital healthcare transformation leads to resilience in terms of quality of life, healthcare accessibility, and the ability to respond to pandemics, and reduce care costs. Similarly, Alenezi et al. [36] assert that with the reforms of the visiting doctor program in the Ministry of Health, several benefits were attained in terms of budget release systems, the development of electronic databases of doctors, and the recruiting system.

Infrastructure

Considering the impact of policy on infrastructure, researchers found that to reach the goals of Vision 2030 in Saudi Arabia, policy and framework adoption is necessary for transformation [48,49]. As a result, Saudi Arabia needs to create sustainable health models. For that reason, private partnership is very important to achieve these goals [50]. According to Al-Kahtani et al. [34] and Alasiri et al. [35], among several hospitals in both private and government sectors, the private sector was more prepared to reform toward NTP. In fact, Alharbi [20] and Rahman et al. [24] asserted that the most reported issue found is the lack of framework adoption.

Another point of view amplifies that digital transformation leads to enhanced infrastructures of NTP [57]. Despite opposing views, the benefits of these transformations are improved access to healthcare, elimination of health inequality, improved population health, and reduced expenditure for government [38,39]. After looking at this from all sides, the transformation must improve the infrastructure of digital technologies [44-46].

People

Saudi Arabian healthcare system’s distribution of physicians and nurses was approximately 10 per 1000 people in 2023. However, the workforce shortage is estimated to be 287,895 and the reform options are limited to increasing working hours to reduce the staff shortage [31]. A lack of human resources in hospitals with the increased turnover, especially among nurses, makes the healthcare system lack sustainability. As a result, the transformation toward creating a better environment in magnet-designated hospitals increases the satisfaction of this specialty improving the overall goal of achieving better transformation [19,21,27].

To illustrate the transformation wariness among healthcare providers, university medical deans revealed that policy knowledge is lacking in the studies curriculum among medical schools and omits the need for formal training to have the capability of healthcare workers with transformation [26,41]. In fact, Ibrahim et al. [51] assert that almost 90% felt they were part of the transformation and 92.2% supported the reform. Similarly, Althumairi et al. [43] found that the healthcare providers were satisfied with their work, and areas of reform in terms of communication and rewards were highly recommended. As a result, the lack of knowledge of the transformation program is considered a critical issue that needs to be addressed for its negative effect [51].

Following a thorough critical review of the literature utilizing the value transformation theoretical framework, the most cited concern was the sustainability and workforce of the healthcare system. In terms of care delivery, the literature was extensive. On the contrary, insufficient studies have been conducted on infrastructure and people. Furthermore, limited information is available on how to assess the transformation, which remains an unaddressed research question. In fact, according to the literature, disagreements among researchers and arguments were found on the use of best strategies for success in NTP. As a result, a significant portion needed to be identified in the field of people and infrastructure. Additionally, international investigation to establish priorities and address challenges must be stimulated, in particular with the NTP sustainability and workforce.

In light of the literature, the review addressed several gaps in terms of human, geographical, methodological, and empirical factors. In addition, this paper summarizes several recommendations and risks that could manage or enhance the healthcare system with the journey of NTP. Furthermore, future research should incorporate expert consultation and more databases and search engines, such as Scopus and CINAHL (Cumulative Index to Nursing and Allied Health Literature), and examine the impact of NTP from the point of view of medical college faculty members, policymakers, managers, and leaders.

## Conclusions

The transformation journey in Saudi Arabia could meet several hurdles. The planning phase was critical. However, measurement and assessment phases to track development could help to interfere with correction toward the success of NTP.

## Appendices

**Table 3:** Joanna Briggs Institute (JBI) quality assessment. Values of 50% or below indicate a low-quality article, between 50% and 69% are considered average quality, and 70% represent the high quality of the article. Yes = “1”; No = “0”; not applicable = “-”.

Reference	1	2	3	4	5	6	7	8	9	10	11	Total	%
[18]	1	1	1	1	0	1	1	1	1	1	1	10 (11)	90.90
[19]	1	1	1	1	0	1	1	1	1	1	1	10 (11)	90.90
[20]	0	1	1	1	1	1	1	1	1	1	1	10 (11)	90.90
[21]	1	1	1	1	1	1	1	1	-	-	-	8 (8)	100
[22]	1	1	1	1	1	1	1	1	-	-	-	8 (8)	100
[23]	0	1	0	1	0	1	1	1	1	1	1	9 (11)	81.81
[24]	1	1	1	1	0	1	1	1	1	1	1	10 (11)	90.90
[25]	1	1	1	1	0	1	1	1	1	1	1	10 (11)	90.90
[26]	1	1	1	1	1	1	1	1	-	-	-	8 (8)	100
[27]	1	1	1	1	1	1	1	1	-	-	-	8 (8)	100
[28]	1	1	1	1	1	0	1	1	-	-	-	7 (8)	87.5
[29]	1	1	1	1	0	1	1	1	1	1	1	10 (11)	90.90
[30]	1	0	1	1	1	1	1	1	1	1	1	10 (11)	90.90
[31]	1	1	1	1	1	1	1	1	-	-	-	8 (8)	100
[32]	1	1	1	1	0	0	1	1	-	-	-	6 (8)	75
[33]	1	1	0	1	1	1	1	1	-	-	-	7 (8)	87.5
[34]	1	1	1	1	1	1	1	1	-	-	-	8 (8)	100
[35]	1	1	1	1	0	1	1	1	1	1	1	10 (11)	90.90
[36]	1	1	1	1	1	1	1	1	-	-	-	8 (8)	100
[37]	1	1	1	1	0	1	1	1	1	1	1	10 (11)	90.90
[38]	1	1	1	1	1	1	1	1	-	-	-	8 (8)	100
[39]	1	1	1	1	0	1	1	1	-	-	-	7 (8)	87.5
[40]	1	1	1	1	0	1	1	1	1	1	1	10 (11)	90.90
[41]	1	1	1	1	0	1	1	1	-	-	-	7 (8)	87.5
[42]	1	1	1	1	0	1	1	1	-	-	-	7 (8)	87.5
[43]	1	1	1	1	1	1	1	1	-	-	-	8 (8)	100
[44]	1	1	1	1	0	1	1	1	1	1	1	10 (11)	90.90
[45]	1	1	1	0	1	1	-	-	-	-	-	5 (6)	83.33
[46]	1	1	1	1	0	1	1	1	1	1	1	10 (11)	90.90
[47]	1	1	1	1	1	1	1	1	-	-	-	8 (8)	100
[48]	1	1	0	1	1	1	1	1	1	1	-	9 (10)	90
[49]	1	1	1	1	1	1	1	-	-	-	-	7 (7)	100
[50]	1	1	1	1	0	1	1	1	-	-	-	7 (8)	87.5
[51]	1	1	1	1	1	1	1	1	-	-	-	8 (8)	100
